# Supplementary material for: Pluripotent Stem Cell-Derived Hepatocytes Inhibit T Cell Proliferation In Vitro through Tryptophan Starvation
Source: Cells. 2021 Dec 22;11(1):24. doi: 10.3390/cells11010024 (PMC8750013; doi:10.3390/cells11010024)
Supplement: Supplementary file 1 [file cells-11-00024-s001.zip › cells-1474229-supplementary.pdf]

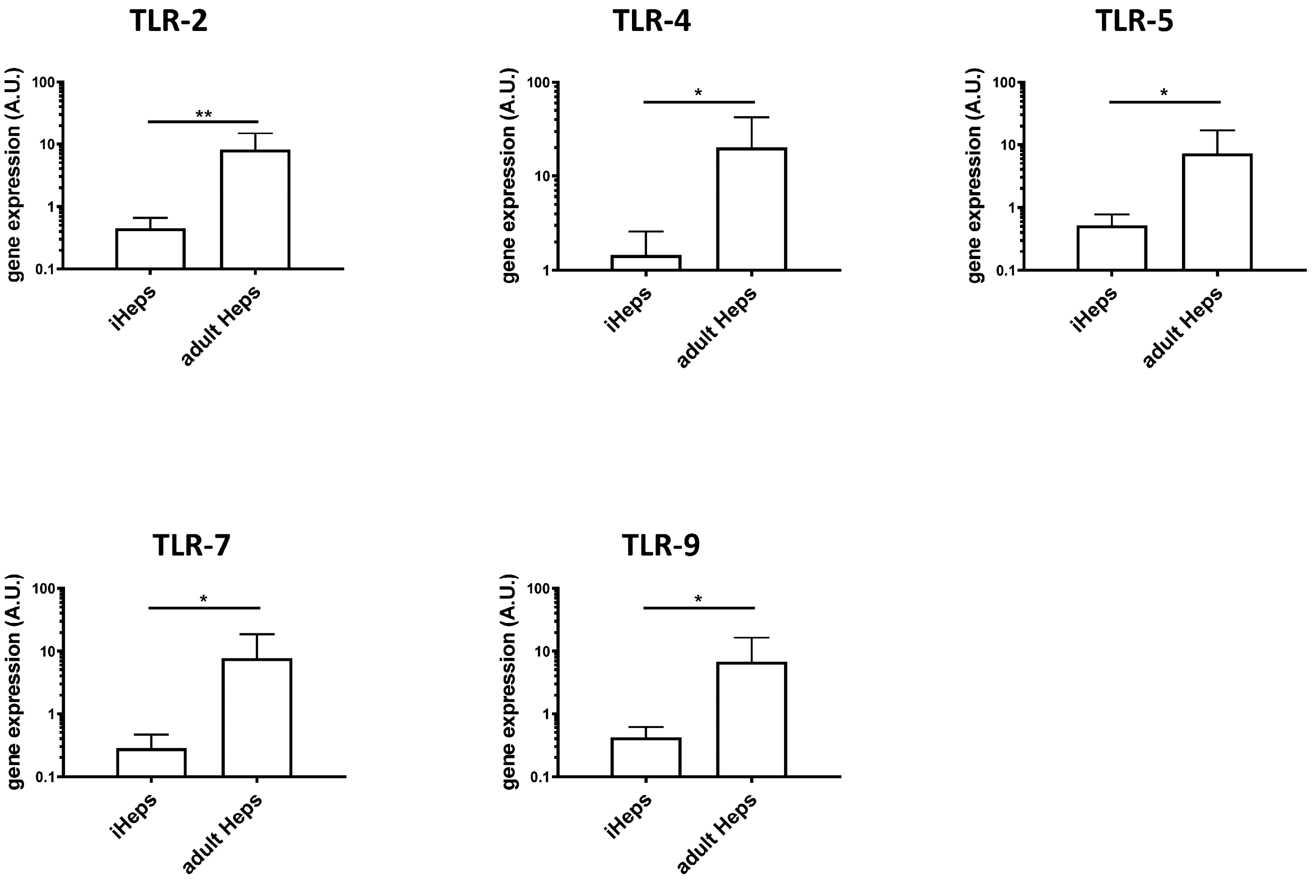

Figure S1: TLR-2, -4, -5, -7, -9 gene expression in HLCs and adult hepatocytes (Heps) evaluated by RT-PCR.

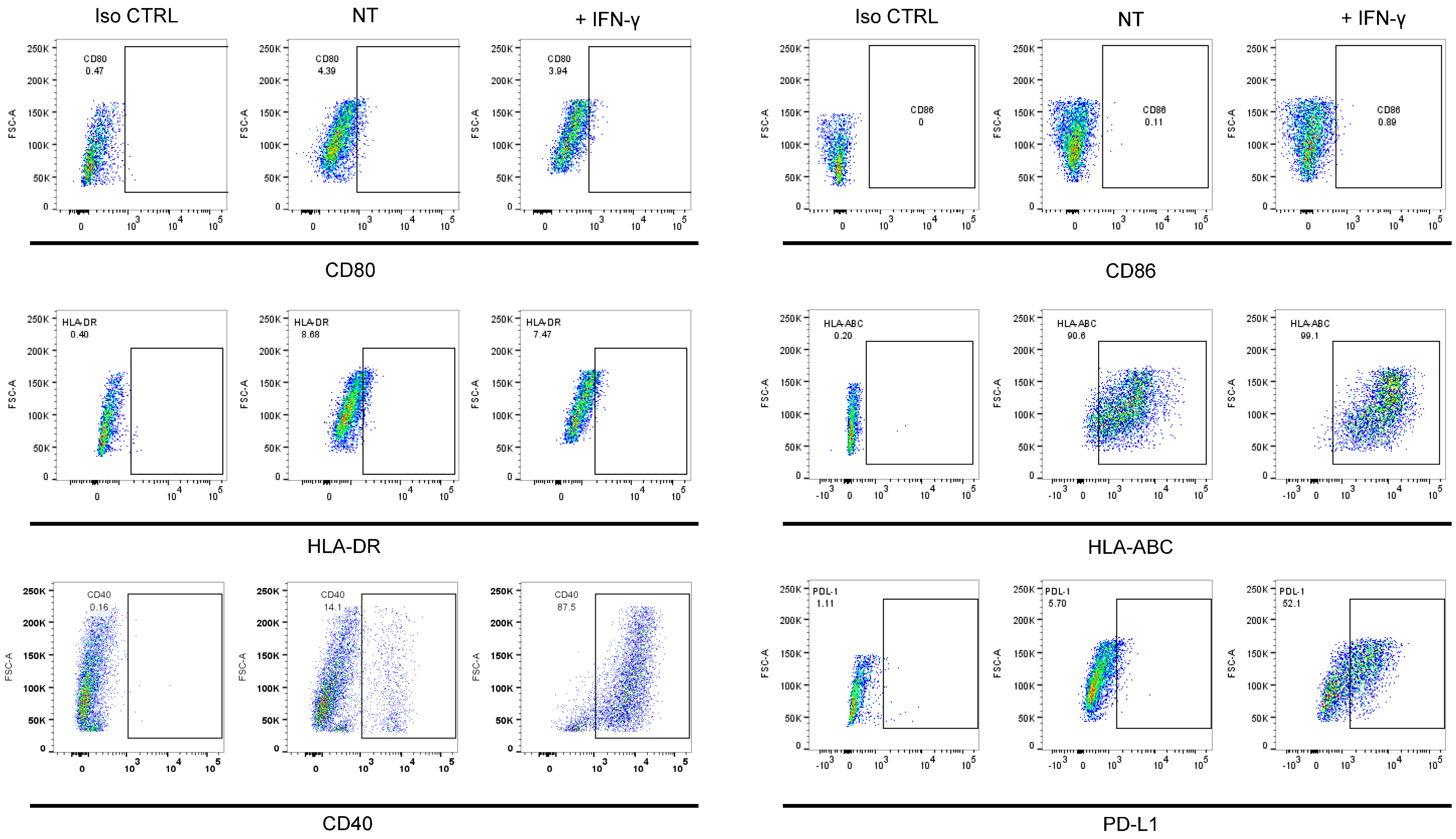

Figure S2: Representative dot plots showing the expression of the indicated markers in NT and INF-γ-pre-treated HLCs.

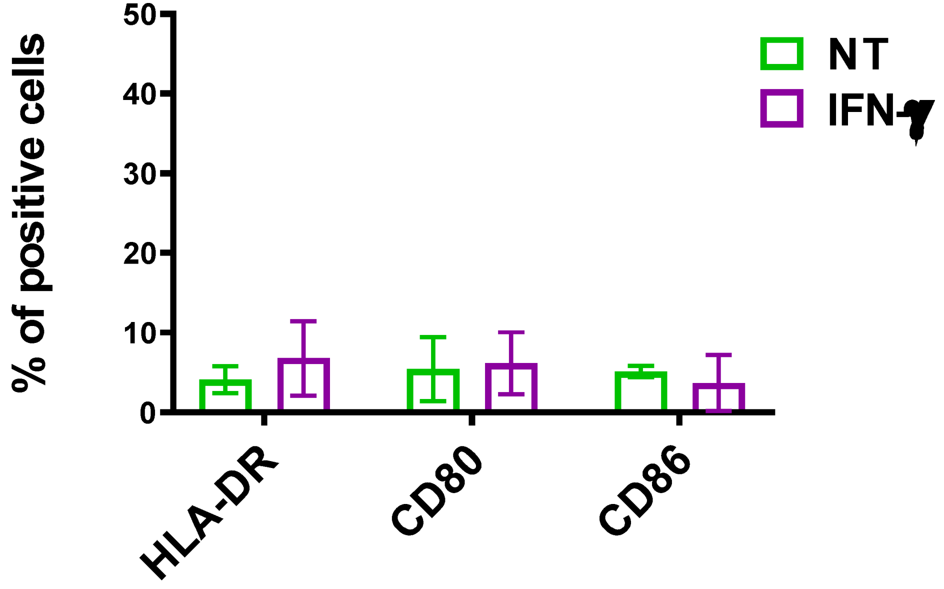

Figure S3: Expression of CD80, CD86 and HLA-DR in NT and INF-γ-pre-treated HLCs. Data are expressed as mean ±SD of 6 independent experiments with one donor per ex-periment.

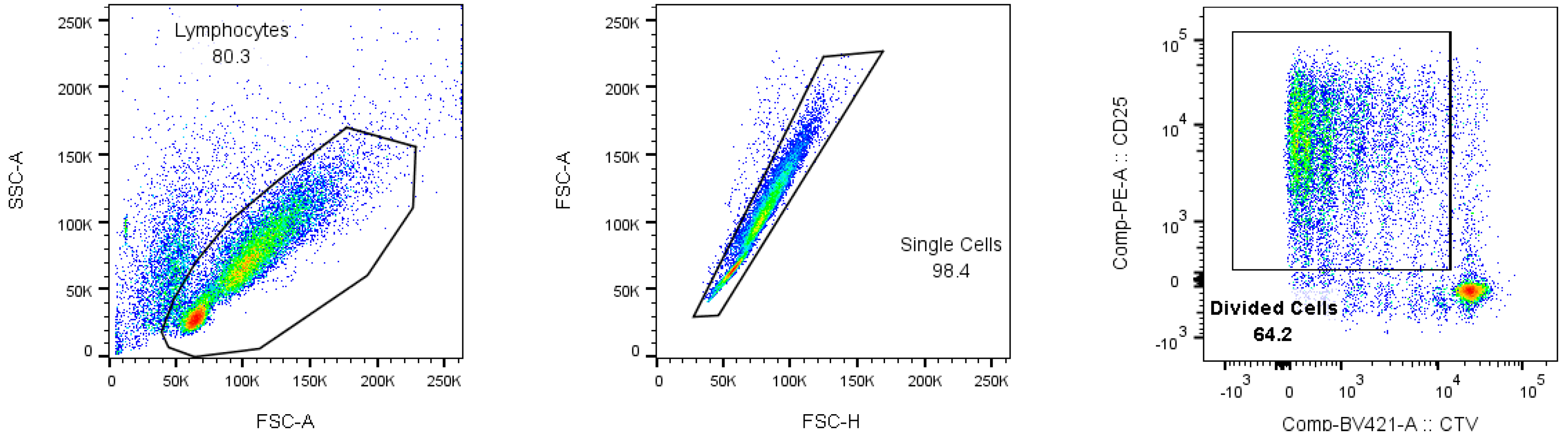

Figure S4: Gating strategy used to evaluate the % of divided cells.

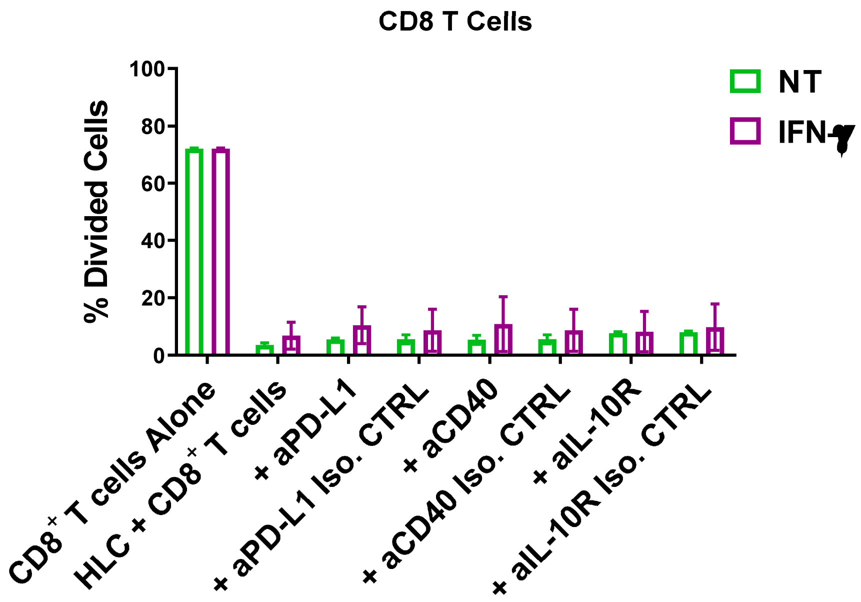

Figure S5: Quantification for percentage of divided CD8-T cells activated with αCD3/CD28 beads and co-cultured with HLCs in the presence of αPD-L1, αCD40 or αIL-10R. Data are expressed as mean ±SD of 3 independent experiments, with one donor per experiment, in duplicate.

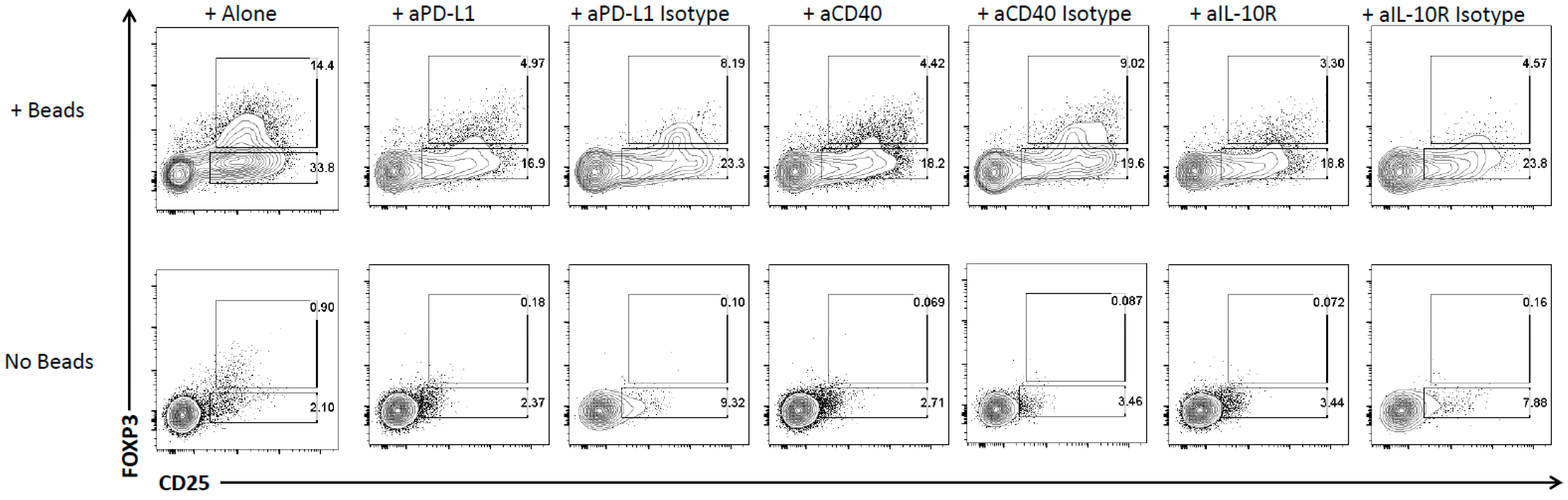

Figure S6: Representative dot plots of FoxP3 expression in CD4+ cells activated with αCD3/CD28 beads and co-cultured with HLCs in the presence of αPD-L1, αCD40 or αIL-10R and the cognate isotype controls.
